# Partner inclusive parenting intervention: Evidence of a culturally adapted low-cost group psychosocial intervention for depressed fathers

**DOI:** 10.1192/j.eurpsy.2023.1781

**Published:** 2023-07-19

**Authors:** M. I. Husain, A. B. Khoso, T. Kiran, M. W. Wan, Z. Zadeh, R. Sattar, N. Khan, I. B. Chaudhry, R. Memon, S. F. Jafri, Z. H. Suhag, K. Lovell, N. Husain, N. Chaudhry

**Affiliations:** 1 The Centre for Addiction and Mental Health, Ontario; 2 University of Toronto, Toronto, Canada; 3 Pakistan Institute of Living and Learning, Karachi, Pakistan; 4 University of Manchester; 5 Manchester Global Foundation, Manchester, United Kingdom; 6 Karachi Medical and Dental College, Karachi, Pakistan

## Abstract

**Introduction:**

Depression is the leading cause of disability worldwide and low and middle-income countries (LMICs) carry over 80% of this disease burden. Attempts have been made to address depression in LMICs, with improvements in the home environment and maternal knowledge. However paternal depression is a neglected and under-researched area. Since maternal depression is associated with depression in fathers there is a need for partner inclusive parenting programs to address parental mental health and improve child outcomes.

**Objectives:**

To evaluate the clinical and cost effectiveness of partner inclusive Learning through play plus (LTP+) intervention in reducing depression in fathers and mothers.

To evaluate the effectiveness of LTP + intervention in improving child outcomes.

To conduct process evaluation and identify challenges in transition to scale up of the intervention across Karachi, Pakistan from the perspective of fathers, mothers, and other stakeholders.

**Methods:**

This is a cluster randomised controlled (cRCT) trial of partner inclusive group parenting program called (Learning Through Play (LTP+) across 18 towns in the city of Karachi. Over 5000 parents (fathers and partners) will participate in the study with a capacity building component of training 4000 Community Health Workers across Pakistan.

**Results:**

This large cRCT will confirm the clinical and cost-effectiveness of LTP+ in reducing depression in parents and improving child outcomes along with the barriers and facilitators to implement the LTP+ group parenting program and the possibilities to roll out the innovation at national level through engagement with policy makers.

**Conclusions:**

Addressing depression in parents is hugely important because of its adverse effects both for child and parents. This low-cost group parenting program will help in scaling up the innovation across health services in Pakistan and other LMICs.

**Disclosure of Interest:**

None Declared

